# Supplementary material for: Multi‐dataset identification of innovative feature genes and molecular mechanisms in keratoconus
Source: J Cell Mol Med. 2024 Sep 19;28(18):e70079. doi: 10.1111/jcmm.70079 (PMC11412914; doi:10.1111/jcmm.70079)
Supplement: Supplementary file 2 — Material S2. [file JCMM-28-e70079-s002.zip › 7.GSEA analysis/3.DBNDD1/KEGG_PENTOSE_AND_GLUCURONATE_INTERCONVERSIONS.html]

Details for gene set KEGG\_PENTOSE\_AND\_GLUCURONATE\_INTERCONVERSIONS[GSEA]

|  || Dataset | DBNDD1.group.cls#h\_versus\_l |
| Phenotype | group.cls#h\_versus\_l |
| Upregulated in class | h |
| GeneSet | KEGG\_PENTOSE\_AND\_GLUCURONATE\_INTERCONVERSIONS |
| Enrichment Score (ES) | 0.5736152 |
| Normalized Enrichment Score (NES) | 1.5475175 |
| Nominal p-value | 0.04329897 |
| FDR q-value | 0.9234659 |
| FWER p-Value | 0.691 |
Table: GSEA Results Summary

  

Fig 1: Enrichment plot: KEGG\_PENTOSE\_AND\_GLUCURONATE\_INTERCONVERSIONS      
 Profile of the Running ES Score & Positions of GeneSet Members on the Rank Ordered List

  

| SYMBOL | TITLE | RANK IN GENE LIST | RANK METRIC SCORE | RUNNING ES | CORE ENRICHMENT || 1 | UGT1A8 | na | 313 | 0.502 | 0.1839 | Yes |
| 2 | UGT2B11 | na | 1393 | 0.345 | 0.2852 | Yes |
| 3 | UGT2B4 | na | 2640 | 0.266 | 0.3515 | Yes |
| 4 | UGT1A6 | na | 2660 | 0.265 | 0.4527 | Yes |
| 5 | XYLB | na | 4038 | 0.212 | 0.4946 | Yes |
| 6 | DHDH | na | 4238 | 0.205 | 0.5677 | Yes |
| 7 | UGT2B28 | na | 6027 | 0.150 | 0.5736 | Yes |
| 8 | DCXR | na | 8230 | 0.099 | 0.5483 | No |
| 9 | RPEL1 | na | 9144 | 0.083 | 0.5540 | No |
| 10 | CRYL1 | na | 11444 | 0.053 | 0.5080 | No |
| 11 | UGT2A3 | na | 13213 | 0.036 | 0.4710 | No |
| 12 | UGP2 | na | 17846 | 0.004 | 0.3390 | No |
| 13 | UGT2B10 | na | 17872 | 0.004 | 0.3397 | No |
| 14 | UGT2B15 | na | 18962 | -0.004 | 0.3100 | No |
| 15 | UGDH | na | 19756 | -0.011 | 0.2913 | No |
| 16 | GUSB | na | 20109 | -0.014 | 0.2865 | No |
| 17 | RPE | na | 20513 | -0.017 | 0.2816 | No |
| 18 | UGT2B7 | na | 24690 | -0.066 | 0.1868 | No |
| 19 | UGT2A1 | na | 28049 | -0.132 | 0.1408 | No |
| 20 | AKR1B1 | na | 28153 | -0.134 | 0.1894 | No |
Table: GSEA details [plain text format]

  

Fig 2: KEGG\_PENTOSE\_AND\_GLUCURONATE\_INTERCONVERSIONS      
 Blue-Pink O' Gram in the Space of the Analyzed GeneSet

  

Fig 3: KEGG\_PENTOSE\_AND\_GLUCURONATE\_INTERCONVERSIONS: Random ES distribution      
 Gene set null distribution of ES for **KEGG\_PENTOSE\_AND\_GLUCURONATE\_INTERCONVERSIONS**
